# Prevalence and predictors of psychological assistance services for older individuals in Turkish society

**DOI:** 10.3389/fpubh.2022.1060845

**Published:** 2023-01-04

**Authors:** Hüseyin Coşkun, Ibrahim Yildiz, Ömer Alkan

**Affiliations:** ^1^Department of Finance, Banking and Insurance, Vocational School of Social Sciences, Bayburt University, Bayburt, Turkey; ^2^Department of Management Information Systems, Faculty of Economics and Administrative Sciences, Ataturk University, Erzurum, Turkey; ^3^Department of Econometrics, Faculty of Economics and Administrative Sciences, Ataturk University, Erzurum, Turkey

**Keywords:** aging, psychological support, Turkey, elderly, binary logistic regression, older individuals

## Abstract

**Background/aim:**

Due to the high contribution of psychological problems to the decline in the comfortable lifestyle of the older individuals, determining the factors that affect individuals' receiving psychological services and searching for solutions based on these factors is one of the primary concerns of national and international decision-makers. This study aimed to identify the factors that affect older individuals' access to psychological assistance services in Turkey.

**Methods:**

In this study, the Turkish Health Research micro dataset, which was conducted by the Turkish Statistical Institute (TURKSTAT) in 2016 and 2019, was employed. In this dataset, the data of 7,252 older individuals were analyzed. Using binary logistic analysis, the factors that are effective for obtaining psychological assistance for the older individuals were identified.

**Results:**

According to the results of the analysis, factors such as gender, education, general health status, disease status, payment of health expenditures, and body mass index affected older individuals who received psychological assistance.

**Conclusion:**

In recent years, there has been a rise in the availability of psychological assistance services for the older individuals. By identifying factors that increase the likelihood of receiving psychological assistance services, this study is expected to contribute to the creation and regulation of psychological assistance services to prevent possible psychological problems that may occur in old age, minimize the problems experienced by individuals, and promote a more comfortable lifestyle.

## 1. Introduction

The aging process is a biological fact, with its dynamics largely beyond human control. Old age refers to the decline in an individual's ability to adapt to an environment beyond his or her control, and chronologically denotes those aged 60 and older (1). Furthermore, aging is a phenomenon that should be evaluated by combining the psychological and biological internal capacity of individuals, the reserves accumulated throughout life, and the composition of social processes. There are biological, psychological, and social factors that affect the aging process of each individual (2). Biologically induced aging is the result of a wide variety of molecular and cellular damage accumulating over time. This results in a gradual decline in physical and mental capacity, an increase in disease risk, and ultimately death (3). People of all ages may experience very common life stressors, such as loss of functional ability due to aging (4). For numerous reasons, older individuals often experience feelings of isolation, depression, and physical inactivity (5). Sixty percent of hospitalized patients over the age of 65 have or will acquire mental health problem, such as dementia, delirium, or depression (6). Therefore, evaluating mental health problems in older individuals is complex due to various problems and diseases seen with physical illnesses (7). Healthy older individuals' psychological problems may be alleviated by participating in social activities (8). Otherwise, exposure to social discrimination may cause psychological problems and reduce individuals' quality of life of individuals (9). Evaluating mental health problems in older individuals is complicated by the variety of problems and co-morbidities associated with physical illness (10).

Mental health is essential for a comfortable life, including the capacity to form relationships, maintain schooling and a career, and make daily decisions (11). Therefore, early treatment is necessary to protect older adults from the negative effects of physical and mental health issues (5, 12). The most prevalent functional disease among older people who experience mental health issues is depression. Depression can be expressed as a change in someone's mood, self-blame, self-punishment requests, and changes in activity levels (13). Depression can cause worse functional disorders in daily life than those with chronic medical conditions, such as lung disease, hypertension, or diabetes. In addition, negative health effects, such as dementia, death anxiety, inability to have children, somatic sickness, cognitive impairment, functional impairment in activities connected to daily life, and lack of social connections, may arise in the older after depression (4, 5, 14–16). Psychological distress, a general unpleasant mood state associated with anxiety or depression, may increase with age (17). Individuals experiencing psychological distress are not open to professional psychological help, and often seek help from individuals without mental health training. However, inadequate or delayed help-seeking behavior is an obstacle to effective treatment (12, 18). As older individuals increasingly receive care in the community as opposed to hospitals, both their physical and social functioning should be evaluated (19). The evaluation of social functions can assist in the development of effective health interventions and policies for the older (20), because weak social support networks pose a threat to the psychological and physical health of the older (21). How older individuals can be treated for psychological problems and how to reach the aged has become a global problem (22, 23). The disagreement between those who provide services in this field centers on how older individuals living in these conditions can be best serve (24).

Globally, the life expectancy of those aged 60 and above is increasing. As a result of decreasing birth rates and rising life expectancy, global populations are aging, and the share of older population in the global population is increasing (3). According to the International Population Report, the average age of the global population has increased at an unpredictably high rate, and corresponding social and demographic shifts have occurred. The report stated that the age group of 60 and over constituted 7% of the world's population and reached 506 million in 2008 (25). The older population is increasing every year. This share, which reached approximately 1 billion at the beginning of 2020, increased to 1.4 billion at the end of 2020. Moreover, it is estimated that by 2030, one out of every six individuals on the planet will be 60 or older, and that number will reach nearly 2.1 billion by 2050 (3, 5). In Turkey, the proportion of the older population to the overall population climbed from 8.2% in 2015 to 9.5% in 2020. According to population projections, it is predicted that the proportion of the older population will be 11.0% in 2025, 12.9% in 2030, 16.3% in 2040, 22.6% in 2060 and 25.6% in 2080, respectively (26).

With the expansion in the world's older population, the psychological problems associated with aging and the health services provided to prevent or solve these problems have been the focus of research and investigation from numerous angles. It is thought that identifying the factors that affect the older in receiving psychological assistance services will contribute to the determination of where psychological support services should be focused, thus allowing the older to live a pleasant life. Studies in this area in Turkey are limited. Therefore, the question addressed in this study is, “What are the factors that are effective in getting psychological assistance services for the older individuals in Turkey?” To address the issue, comprehensive data collection was used to model the factors affecting the older individuals' receipt of psychological assistance services in Turkey.

## 2. Methods

### 2.1. Data source

In this study, the Turkish Statistical Institute's 2016 and 2019 micro datasets for the Turkey Health Survey was used. The Turkey Health Survey is one of the most thorough studies conducted to produce many indicators that cannot be derived from administrative records in the field of health and to create a data source for decision-makers and researchers on this subject. The scope of the research was the households in all settlements within Turkey's borders. The stratified two-stage cluster sampling method was used in the sample design of the Turkey Health Survey (27, 28). The stratified two-stage cluster sampling method is a sampling technique used to obtain an efficient estimation by selecting certain elements in selected clusters. Stratified two-stage cluster sampling has been widely used for effective estimations due to time and cost (29). The rural-urban distinction was used as an external stratification criterion (Settlements with a population of 20,000 and below are considered rural, and settlements with a population of 20,001 and above are considered as urban). The first stage sampling unit was randomly selected blocks from clusters (blocks) containing an average of 100 addresses; the second stage sampling unit was the household addresses selected randomly from each selected cluster (27, 28). Data from a total of 7,252 older individuals, 3,250 men and 4,002 women aged 60 and over, were used, and all data were included in the analysis since there were no missing observations.

### 2.2. Outcome variable

In the Turkey Health Survey, the following questions were asked about individuals receiving psychological assistance services: “Have you seen a psychologist for yourself in the last 12 months?” “Have you seen a psychotherapist for yourself in the last 12 months?” “Have you seen a psychiatrist for yourself in the last 12 months?” Older individuals participating in the study were considered to have received psychological assistance if they answered “yes” to one or more of the questions listed above. As a result, the dependent variable of the study was the status of receiving psychological assistance service for the older who received the code 1 if they had seen a psychologist, psychotherapist, or psychiatrist in the last 12 months, and 0 if they had not visited any of them.

### 2.3. Independent variables

In this study, which examined the factors that affect older individuals receiving psychological assistance services, some sociodemographic and health status questions posed to the survey participants were examined, and some variables that were predicted to be effective were included in the model. The variables included in the model were survey year (2016, 2019), age (60–64 years, 65 years and older), education level (illiterate or not completed school, primary school, elementary school, high school, or university), general health status (poor or very poor, moderate, and good or very good), disease status, and body mass index (thin, normal weight, overweight, obese, and extremely obese).

While the questionnaire did not ask about any physical or mental illness, the question “Do you have a disease or health problem that has lasted for 6 months or longer or is expected to last?” was asked (yes or no). Regarding the difficulty in paying for healthcare, the following questions were asked: “In the last 12 months, have you ever needed medical care but could not afford it?”; “In the last 12 months, have you been unable to afford the prescribed medication you needed?”; and “In the last 12 months, have you ever needed mental treatment (by a psychiatrist, etc.) but could not afford?” If the answer was “yes” to at least one of these questions, the participant was evaluated as having difficulty paying for healthcare.

All of the variables addressed here are categorical variables with either ordinal or two-state scales. Ordinal and nominal variables were defined as dummy variables so that the effects of the categories of all variables to be included in the binary logistic regression model could be observed (30, 31).

### 2.4. Statistical analysis

Survey statistics in Stata 15 (Stata Corporation) were used to account for the complex sampling design and weights (32). Weighted analysis was performed (33, 34). First, frequencies and percentages of older individuals were determined according to their psychological support status. The Chi-square independence test was used to examine the relationship between psychological support status and independent variables. Then, risk factors that are effective in receiving psychological support were determined using the binary logistic regression analysis (35, 36).

## 3. Results

### 3.1. Characteristics of study participants

This section interprets the frequency and percentages of the independent variables associated with the model to be developed. The chi-square test statistics are presented in Table 1, together with the factors that affect the older individuals' receipt of psychological assistance services.

**Table 1:** Prevalence of factors affecting older individuals' receiving psychological assistance services and chi-square test statistics.

**Variables**	**Status of receiving physiological assistance service**		***n* (%)**	**χ^2^**	***P***
	**No**	**Yes**			
**Year**					
2016	3,490 (51)	167 (40.9)	3,657 (50.4)	15.595	0.000^a^
2019	3,354 (49)	241 (59.1)	3,595 (49.6)		
**Gender**					
Male	3,123 (45.6)	127 (31.1)	3,250 (44.8)	32.751	0.000^a^
Female	3,721 (54.4)	281 (68.9)	4,002 (55.2)		
**Age**					
60–64 years	2,138 (31.2)	144 (35.3)	2,282 (31.5)	2.936	0.087^c^
65 and over	4,706 (68.8)	264 (64.7)	4,970 (68.5)		
**Education**					
Illiterate or not completed a school	2,555 (37.3)	143 (35)	2,698 (37.2)	5.412	0.248
Primary school	2,980 (43.5)	181 (44.4)	3,161 (43.6)		
Elementary school	380 (5.6)	19 (4.7)	399 (5.5)		
High school	477 (7)	27 (6.6)	504 (6.9)		
University	452 (6.6)	38 (9.3)	490 (6.8)		
**General health status**					
Very good or good	1,904 (27.8)	50 (12.3)	1,954 (26.9)	63.173	0.000^a^
Moderate	2,968 (43.4)	180 (44.1)	3,148 (43.4)		
Poor or very poor	1,972 (28.8)	178 (43.6)	2,150 (29.6)		
**Disease status**					
Yes	5,686 (83.1)	387 (94.9)	6,073 (83.7)	39.199	0.000^a^
No	1,158 (16.9)	21 (5.1)	1,179 (16.3)		
**Difficulty in health care payments**					
Yes	572 (8.4)	69 (16.9)	641 (8.8)	34.966	0.000^a^
No	6,272 (91.6)	339 (83.1)	6,611 (91.2)		
**Body mass index**					
Thin (Under 18.5 kg/m^2^)	99 (1.4)	4 (1)	103 (1.4)	15.379	0.004^a^
Normal weight (18.5–24.999 kg/m^2^)	1,942 (28.4)	83 (20.3)	2,025 (27.9)		
Overweight (25–29.999 kg/m^2^)	2,738 (40)	175 (42.9)	2,913 (40.2)		
Obese (30–39.999 kg/m^2^ and over)	1,906 (27.8)	138 (33.8)	2,044 (28.2)		
Extremely obese (40 kg/m^2^ and over)	159 (2.3)	8 (2)	167 (2.3)		

^a^p < 0.01, ^c^p < 0.10.

According to the findings presented in Table 1, 44.8% of the older individuals were male, and 55.2% were female. Individuals aged 65 and above constituted 68.5% of the study sample. Of the study's participants, 37.2% were either illiterate or had not completed high school, while 6.8% were university graduates. Those with good or very good general health constituted 26.9% of the study population, while 29.0% had poor or very poor health. Older individuals with health problems lasting 6 months or longer constitute 83.7% of the study. Older individuals, who could not afford medical care, prescribed medication, or psychological support in the last 12 months constituted 8.8% of the study. According to the body mass index, 1.4% of the sample population was underweight, 40.2% was overweight, and 2.3% was obese. The chi-square test statistics of all variables except education were found to be significant.

### 3.2. Multivariate analyses

In this study, the binary logistic regression model was used to determine the factors that affect whether or not the older obtain psychological assistance services. The estimated model results and the marginal effects of the factors affecting the psychological support status of the older are shown in Table 2.

**Table 2:** Estimated model results and marginal effects on factors effective in receiving psychological assistance services for older individuals.

**Variables**	**β**	**Std. error**	**95% CI**		**ME (%)**	**Std. error**	**VIF**
			**Lower**	**Upper**			
**Year (reference: 2016)**							
2019	0.412^a^	0.125	0.167	0.657	38.95^a^	0.118	1.020
**Gender (reference: male)**							
Female	0.612^a^	0.137	0.344	0.881	57.97^a^	0.130	1.240
**Age (reference: 60–64 years)**							
65 years of age and over	−0.207	0.131	−0.464	0.049	−19.52	0.123	1.080
**Education (reference: university)**							
Illiterate or not completed a school	−1.249^a^	0.233	−1.706	−0.792	−115.26^a^	0.209	4.62
Primary school	−0.780^a^	0.210	−1.193	−0.367	−70.80^a^	0.186	4.32
Elementary school	−0.739^b^	0.354	−1.434	−0.044	−66.97^b^	0.324	1.72
High school	−0.673^b^	0.283	−1.227	−0.119	−60.84^b^	0.256	1.89
**General health status (reference: very good or good)**							
Moderate	0.620^a^	0.202	0.225	1.016	59.46^a^	0.194	1.86
Poor or very poor	0.998^a^	0.217	0.573	1.422	94.72^a^	0.207	2.08
**Disease status (reference: no)**							
Yes	0.733^b^	0.290	0.165	1.301	70.13^b^	0.281	1.36
**Difficulty in health care payments (reference: no)**							
Yes	0.369^b^	0.164	0.046	0.691	34.52^b^	0.153	1.04
**Body mass index [reference: normal weight (18.5–24.999 kg/m** ^2^ **)]**							
Thin (Under 18.5 kg/m^2^)	−0.463	0.568	−1.577	0.651	−44.72	0.552	1.04
Overweight (25–29.999 kg/m^2^)	0.390^b^	0.164	0.068	0.712	36.98^b^	0.156	1.48
Obese (30–39.999 kg/m^2^ and over)	0.421^b^	0.175	0.078	0.764	39.89^b^	0.166	1.52
Extremely obese (40 kg/m^2^ and over)	−0.048	0.441	−0.913	0.817	−4.59	0.423	1.09

^a^p < 0.01, ^b^p < 0.05.

When Table 2 is examined, it is seen that the variables of survey year, gender, education level, general health status, disease status, inability to pay for health care and body mass width (overweight, obese) are significant. Additionally, multicollinearity between the model's independent variables was investigated. Those with variance inflation factor (VIF) values of 5 or above are thought to generate significant multicollinearity, and those with VIF values of 10 or higher cause a high degree of multicollinearity (37, 38). According to the VIF results presented in Table 2, none of the variables are responsible for the multicollinearity problem between the variables.

According to the binary logistics model presented in Table 2, other variables being constant, an older individual who participated in the study in 2019 was 39% more likely to receive psychological assistance services than an older individual who participated in the same study in 2016. Older women are 58% more likely to receive psychological assistance than older men. Older individuals over the age of 65 who were illiterate or had not completed high school were 115% less likely to receive psychological assistance than those with a university degree. Primary school graduates were 71% less likely than university graduates to receive psychological assistance. Graduates of elementary schools were 67% less likely to receive psychological assistance than university graduates. Those with a high school diploma were 61% less likely to receive psychological assistance than those with a university degree. An older individual with a moderate general health status was 60% more likely to receive psychological assistance than an older individual with a good or very good health status. An older individual with a poor or very poor health status was 95% more likely to receive psychological assistance services than an older individual with a good or very good health status. Older individuals with health problems lasting 6 months or longer, or expected to last, were 70% more likely to receive psychological assistance than those without health problems. In the previous 12 months, those who were unable to pay for medical care, prescription medication, or psychological support were 35% more likely to receive psychological assistance than those without financial difficulties. Overweight older individuals were 37% more likely to receive psychological assistance than normal-weight older individuals. Obese older individuals were 40% more likely to receive psychological assistance services than normal-weight older individuals.

## 4. Discussion and conclusions

Binary logistic regression analysis is used to analyze the effective factors for older individuals in Turkey to receive psychological assistance services. The study included older individuals living in Turkey, and the data obtained for two different years. According to the findings of the study, the probability of receiving psychological assistance services for older individuals has increased in recent years. Women received greater psychological assistance than men as they aged. A high degree of education improved the likelihood of older individuals receiving psychological assistance. The worse of the general health status of older individuals, the less likely they were to receive psychological assistance services. Exposure to disease for at least 6 months increased the probability of seeking psychological assistance. Older patients with problems paying for healthcare were more likely to receive psychological assistance. Those who were overweight or obese were more likely to seek psychological assistance services.

According to the findings, older individuals who participated in the survey in 2019 were more likely to receive psychological assistance than those participated in 2016. With the rising participation of women in the workforce and the restructuring of family structures into nuclear families, care for the older has become increasingly challenging in recent years. Studies on the older who do not live with their families focus mostly on issues such as treatment, meeting their daily needs, and improving their social relationships. However, psychological services appear to have flaws (39). In addition to the persistence of these problems in the past, it is thought that the rapid increase in the proportion of the older population has reduced the likelihood that older individuals will receive psychological assistance over time.

According to the results of the estimation, older women in Turkey were more likely to receive psychological assistance services than older men. In a similar study conducted in Turkey, it was determined that the rate of married persons declined with age, the rate of those who lost their wives and became widowed in old age increased, and widowhood produced significant psychological problems, such as loneliness (40). A study conducted on older women revealed that women were removed from economic and social life due to their longer average life expectancy and traditional gender roles; therefore, older women experienced more economic and psychosocial issues today (41). In many countries, women have lower social standing and earn less than men. It has been stated that this poses a significant health risk (42). Another study revealed that gender had a significant impact on the degree of depression among the older adult groups participating in the survey and that women's average mental health score was significantly lower than that of the men (43). In addition to the findings of these studies conducted in Turkey, it can be stated that the higher social pressure on women in underdeveloped regions, the low rate of women's workforce participation, and the fact that a large portion of the domestic workload falls on women are effective in increasing the likelihood of older women receiving psychological support.

One of this study's noteworthy findings is related to the education. Older individuals who were illiterate or did not complete high school, primary school graduates, elementary school graduates, and high school graduates were less likely to obtain psychological assistance services than older individuals who had graduated from university. It was observed that the likelihood of receiving psychological support increased with higher levels of education. The expectations that people have of life will rise along with the amount of education. The inability to meet expectations arising from sociological, physiological, and psychological changes that occur in old age may reveal the need for psychological support in older individuals. Older individuals generally receive support from the community rather than professional units (19). However, individuals with a high level of education know how to reach institutions where they can receive psychological support. In addition, unlike those living in rural areas, where health services are lacking, people living in urban areas, where the level of education is higher, are able to access health services more easily (44, 45). It is thought that the ease of accessing health services will increase the probability of receiving psychological support for those with a high education level compared to those with a low level of education.

It has been determined that older individuals with a moderate general health status are more likely to receive psychological assistance than those with a good or very good general health status, and those with a poor or very poor general health status are more likely to receive such services than those with a moderate general health status. When disease condition was considered, it has been observed that older individuals with health difficulties projected to last 6 months or more are more likely to receive psychological assistance than those without health issues. According to some studies in the literature, individuals with chronic diseases are unable to sustain self-care, cannot perform daily living activities, require the assistance of another person, and have lower levels of adaption to old age (46–49). On the basis of these findings, it is thought that negative factors, such as movement restrictions and loss of ability due to chronic diseases, cause negative changes in the psychological status of older individuals, and therefore, increase the possibility of receiving psychological assistance services.

When the variable of insolvency in health was considered, it was determined that the older who could not afford medical care, taking prescribed medication, or psychological support in the past 12 months were more likely to receive psychological assistance services than the older who did not have payment difficulties. The fact that older individuals who had lost their income due to retirement or who did not have a regular income had difficulty paying the contribution fees that must be paid for individual treatment and medical supplies may increase their health concerns, which may lead to psychological problems and increase their likelihood of receiving psychological assistance services.

It was observed that older individuals who were overweight and obese were more likely to receive psychological assistance services than those with normal weight. These findings confirmed research demonstrating that overweight and obesity may be associated with a variety of psychological issues (50, 51). Especially in older individuals, the issue of excessive weight can result in movement difficulties and a sense of personal inadequacy. It is thought that older individuals who cannot overcome these problems may need psychological support.

In Turkey, private and public older care organizations and older care centers belonging to non-governmental organizations provide care and rehabilitation services to older people who can meet their own needs or need private residential care. However, services are provided for those who apply for older care in Turkey, and even if they need the service, other older people who do not apply or cannot access the service cannot be reached. There is no common standard in the services provided, and it cannot be disseminated in all local governments. In addition, preventive and protective services in older care are insufficient, and lifelong learning studies for the older have not been developed enough to raise awareness among the older individuals and their relatives (52). It is of great importance for both society and the family to build the social support programs required to improve the quality of life of the older population, which will constitute a substantial portion of the future Turkish population, and to ensure that they live an active life.

In addition, it is important to determine the reasons why the older individuals seek psychological support, as well as to establish a common standard in the services provided in Turkey, to identify individuals who need psychological support services, and to expand service organizations in all local governments. Considering that the factors affecting the mental health of the older individuals may differ in different countries, revising the practices in different countries by integrating cultural features or creating new practices would be of great benefit to the mental health development of the older individuals. This study is expected to contribute to the creation and regulation of psychological support services to prevent possible psychological problems that may occur in old age, to minimize the problems experienced by older individuals, and to promote a more comfortable lifestyle by identifying factors that increase the likelihood of receiving psychological assistance services. Furthermore, in future research on the psychological problems of old age and the elimination of these problems, the different econometric models and variables used in the findings of this study can be applied.

To protect and improve community mental health, the systems of countries with high happiness rates should also be examined. To protect the mental health of the community, resources should be increased, and a service model adopted by the community should be created. Cross-sectoral cooperation is necessary for the fight against mental health problems. For this, intersectoral cooperation should take place. To increase the appropriate counseling outreach skills of the personnel who will work in mental health services, there should be compulsory training that will improve their communication and counseling skills (53).

Implementing initiatives that increase hope for the older individuals, strengthen emotional support, and provide quality health care may benefit the lives of those who have retired and deserve a peaceful, comfortable old age. Telephone and video conference calls for older individuals who cannot meet with their loved ones during this period can help increase their life satisfaction. In addition, emotional support and active social participation should be provided to older individuals to promote their happiness.

### 4.1. Limitations of the study

This study has several limitations. First, the data in this study were secondary data. The variables required for statistical analysis consisted of the variables in the dataset. However, some variables, such as occupation and homeownership, that were not included in the data set could not be included in the analysis. Second, because the data are cross-sectional, the definite causal relationship between verbal violations and related socio-economic factors cannot be inferred (54). Third, the data on questions asked about receiving psychological assistance services to individuals were the individuals' own answers. Therefore, the data obtained in this data collection method may be biased.

## Data availability statement

The data analyzed in this study is subject to the following licenses/restrictions: The data underlying this study is subject to third-party restrictions by the Turkey Statistical Institute. Data are available from the Turkish Statistical Institute (bilgi@tuik.gov.tr) for researchers who meet the criteria for access to confidential data. The authors of the study did not receive any special privileges in accessing the data. Requests to access these datasets should be directed to bilgi@tuik.gov.tr.

## Author contributions

HC, IY, and ÖA: design and development of the study and writing-original draft. HC and ÖA: conceptualization, methodology, formal analysis, and data curation. HC: editing. HC and IY: supervision. ÖA: software and visualization. ÖA and IY: writing - review and editing. All authors contributed to the article and approved the submitted version.
